# Effect of edible chitosan film enriched with anise (*Pimpinella anisum* L.) essential oil on shelf life and quality of the chicken burger

**DOI:** 10.1002/fsn3.544

**Published:** 2017-12-01

**Authors:** Vahid Mahdavi, Seyed Ebrahim Hosseini, Anousheh Sharifan

**Affiliations:** ^1^ Department of Food Science and Technology Science and Research Branch Islamic Azad University Tehran Iran

**Keywords:** Anise essential oil, antimicrobial, chicken burger, chitosan, shelf‐life

## Abstract

In this study, the effect of chitosan film (CF) with different concentrations of anise essential oil AEO (0, 0.5, 1, 1.5 and 2%) on the quality of chicken burger during chilled storage (4 + 1°C) were examined over a period of 12 days. For this purpose, at the first, the physical and mechanical properties of the produced films were studied. Then, the chicken burger was covered with the produced films. Different treatment were analyzed by biochemical properties such as moisture and thiobarbituric acid, bacteriological properties such as total viable counts and total psychrotrophic counts, *Pseudomonas aeruginosa*,* Staphylococcus aureus*, and *Escherichia coli*. The results of this study showed that adding AEO improved the properties of CF, the moisture, solubility, and water vapor permeability decreased in these films. By increasing the concentration of AEO the tensile strength and elasticity of film were increased. CF with AEO, delayed lipid oxidation in chicken burger and improved the chemical properties of chicken burger. Also, microbial spoilage in these samples decreased significantly (*p* < .05) compared to the control sample. AEO at 2% in all experiments had better results than other treatments (*p* < .05), and the AEO (1.5% and 2%) had acceptable biochemical, bacteriological attributes up to end of storage, and these treatments could reduce the population of pathogen bacteria below the acceptable level from day 3 until the end of the storage period. Sensory score of the treatment containing AEO at 1.5% was higher than the sensory score of AEO at 2%. Overall, the results of this study showed that the use of CF with AEO as a natural preservative increased the shelf life of meat products. Considering the relatively similar anti‐oxidation and antimicrobial effect of AEO at 1.5 and 2% and also economic aspects, optimum dose for AEO could be 1.5% in the film.

## 1 INTRODUCTION

With the increase in population and the emergence of new phenomena such as the development of cities, the establishment of factories and, consequently, the employment of family members, to provide livelihood and relative welfare, the need for ready‐made foods has become a necessity and expanded to some extent that these foods are an integral part of people's lives today, while chicken products are of particular importance (Luiz, Moreira, Correa Ede, & Falcao, 2004). One of the products of chicken meat is frozen raw chicken burger. Meat keeping is limited due to its biological composition, even in glacial conditions, and it quickly receives microbial and chemical contamination, which can, in addition to health hazards, cause undesirable changes in its qualitative characteristics such as taste and smell, color, texture and a reduction in nutritional value and, finally, a loss of shelf life (Davidson & Zivanivic, 2003). Oxidative spoilage causes unpleasant smell, undesirable flavor changes, eventually a change in the nutrient structure and a decrease in the nutritional value of the product, while microbial spoilage and contamination lead to serious risks to the health of the consumer. Therefore, the use of appropriate substances with antibacterial and antioxidant activity is useful and necessary to improve the quality and increase the shelf‐life of the meat while preventing economic losses. Antioxidants have been used for many years as additives in food. In fact, antioxidants, by reducing the rate of fat oxidation, increase the shelf‐life of foodstuffs and improve the stability of lipids and lipid foods, thereby preventing the loss of sensory characteristics and their nutritional value (Burt, 2004). Today, the use of natural antioxidants, such as spices and plants, as an alternative to synthetic antioxidants, is highly recommended for food preservation (Sakanaka, Tachibana, & Okada, 2005).

The *Pimpinella anisum* L is from the Umbelliferae family, a herbaceous annual plant, the most part of which is its green, pear‐shaped and small fruits that are found in Turkey, Iran, India, Egypt and many other tropical regions. This plant is used as a flavoring in the food and pharmaceutical industries (Al‐Bayati, 2008; Gulcin et al., 2003). Based on the research, anise essential oil contains anothole, eugenol, methylchavicol, anisaldehyde and estragole and anothole has been identified as an effective ingredient in anise essential oil (Abdel‐Reheem & Oraby, 2015).

According to what stated, the use of natural ingredients in food sources can be a good alternative to artificial preservatives, but restricts the cost of using and other problems, such as odor intensity, potential toxicity, and uncontrolled release of these substances into food products and also inactivation of food. One strategy to reduce the dose of essential oil while maintaining their effectiveness can be adding these natural compounds to edible coatings and films (Sánchez‐González, Vargas, González‐Martínez, Chiralt, & Cháfer, 2011). Chitosan is a biopolymer that has many uses in the food industry and is the second most natural polymer in nature after cellulose. The polysaccharide has functional properties such as microbial, fungicidal and antioxidant properties, and also has properties such as compatibility with environment, biodegradation, nontoxicity and various physical and chemical properties. Other functional properties of chitosan are the ability to form film, adhesion properties, adsorption, refinement and as a dietary fiber used in food, medicine, pharmacy, dyeing, textiles, cosmetics, and so on. A wide range of microorganisms including gram‐positive bacteria, gram‐negative bacteria and molds are sensitive to chitosan (Vásconez, Flores, Campos, Alvarado, & Gerschenson, 2009).

According to the stated contents, the study aimed to investigate the effect of edible Chitosan film containing anise essential oil (AEO) on microbial and chemical properties of chicken burger kept at refrigerated (4 ± 1°C) temperature.

## 2 METHODOLOGY

### 2.1 Preparation and analysis of AEO

Seeds of anise were prepared from the Institute of Forestry and Rangeland Research in Kashan. After confirmation of scientific name by the Institute of Pharmacology, essential oil seeds were powdered using mixture and 150 g of powder was mixed with one liter of distilled water and the extraction was done for 3 hr using sodium sulfate and the essential oil was stored in dark containers until the test was carried out at 4°C (Al‐Bayati, 2008). Identification of compounds was done using various parameters, such as mass spectrum studies, and comparison of these spectra with standard compounds and information available in the library of the GC/MS machine (Adams, 2007). The relative proportions of each of the constituents of the essential oil according to its curved surface in the GC chromatogram were obtained by normalizing the surface and ignoring the response coefficients.

### 2.2 Determination of minimum growth inhibitor concentration (MIC) of anise essential oil

#### 2.2.1 Used *microorganisms*

In this study, three microorganisms were used: *Salmonella typhimurium* ATCC14028, *Staphylococcus aureus* ATCC 65138, and *Escherichia coli* O157: H7. The microorganisms were collected from the Iranian Scientific and Industrial Research Organization. At first, bacteria were cultured in a Muller‐Hinton Agar medium for 18 hr at 37°C. The re‐culture was then taken from the first culture and kept at 37°C for 18 hr. The second culture was mixed to form a stoic with a ratio of 1–5 with sterile glycerin. In 100 μl volumes, It was kept at −20°C in eppendorf tubes, in 100 μl volumes. (Hosseinzadeh et al., 2011).

#### 2.2.2 Determination of the minimum inhibitory growth concentration by microdilution method

Subsequent concentrations of the anise essential oil (100, 125, 150, 175, 200, etc.) were prepared in Muller‐Hinton medium containing 5% Dimethyl Sulfoxide. In this method, plates of 96 houses were used with round holes and 300 μl volume. Then, 100 μl of essential oil was transferred to each well and 20 μl of bacterial suspension was added. The contents of each well were mixed for 2 min by the Shaker‐equipped Plate Reader. Then, light absorption was read using a plate reader at zero and at 630 nm length. Plates were incubated at 35°C for 24 hr and after completing the incubation, cloudiness or lack of cloudiness were observed in the wells and light absorption was read using Plate Reader at the wavelengths (Hosseinzadeh et al., 2011).

### 2.3 Preparation of chitosan‐based films

Chitosan powder (Sigma Aldrich‐America) with high molecular weight was dissolved in 1% acetic acid to give a 2% solution. The resulting solution was stirred at room temperature for one night and was filtered using filter paper No. 3 after addition of glycerol (0.5 ml per g of chitosan) as a plasticizer and tween 80 as an emulsifier for 30 min at room temperature, then the pH of the solution was adjusted to 8.5 using zinc. After adding the desired concentrations of AEO (v/v% 2, 1.5, 1, 0.5, and 0) was homogenized using homogenizer at 12000 g for 2 min and films were molded in Teflon‐coated dishes and dried for 36 hr at 25 ± 2°C. Prepared films were stored for 48 hr before the test in a desiccator containing sodium bromide (25 ± 2°C and 50 ± 2% RH). (Moradi, Tajik, Razavi Rohani, & Oromiehie, 2011).

### 2.4 Measurement of physical properties of films

#### 2.4.1 Measuring film thickness

The thickness of the samples was measured with a digital micrometer (0.001 mm, Mitutoyo made in Japan). Measurements were repeated at five points of each sample. The average thickness was calculated and used to determine the tensile strength and permeability to water vapor (ASTM, 1996).

#### 2.4.2 Measuring the moisture content of the films

The film pieces were cut in 3 × 3 mm and each was weighed. The measured value was placed as the initial weight. Sample pieces were then placed in an oven at 90°C until the final dry weight. Samples were then weighed and the value was considered as dry weight (Hosseini, Razavi, & Mousavi, 2009).

#### 2.4.3 Measuring water vapor permeability of the films

To carry out this experiment, ASTM No. 96 E was used (ASTM, 1996). Water was poured into the cell permeability measurement for testing. Afterwards, the cell surface was coated with molten paraffin. Cells were placed inside the desiccators containing silica gel. Water at 25°C produces 100% moisture. The difference in humidity on the two sides of the coating at 25°C makes the gradient of a steam pressure equal to 2.337*10^3^ psi. Changes in cell weight over time were measured using a digital scale with a precision of 0.0001 g. In all samples, by drawing the cell weight change curve relative to time, a straight line (*R* ^2^ > .99) was obtained. The water vapor transmission rate was equivalent to the slope of the resulting lines divided by the cell surface and was obtained from the following equation: cell surface was 0.00287 m^2^.

Cell surface/Line gradient=Water vapor transfer rate

#### 2.4.4 Measurement of film solubility in water

After weighing, the film pieces were put into 50 cc of distilled water and stirring was done for 6 hr at a temperature of 25°C. Then the film and water mixture was filtered in a filter paper that has already reached constant weight and was accurately weighed. The filter paper was placed at 110°C with the sample to reach constant weight. The solubility of films in water is calculated as follows: (Hosseini et al., 2009).

Solubility percentage=AB/C×100

A = film initial dry weight; B = dry film weight after immersion; C = film initial dry weight.

### 2.5 Measurement of mechanical properties of films:

Stretching experiments were performed using the Instron Universal Testing Machine. The covers were cut in rectangles of 9 × 1 cm. The distance between the jaws was 5 cm and the speed of the jaws was 30 mm/min. Factors including peak force at tearing point, percentage of increase in length at tearing point (change in sample length divided by initial multiplication factor of 100) were achieved using D882‐91 method (American Test and Materials Association) from force curves according to deformation. The tensile strength of the coatings was calculated from the following equation (ASTM, 1996).

$$\begin{array}{cc} (\text{Coating thickness}\times \text{Width of coating}/\\ \text{maximum force at tearing point})= & \text{Tensile strength} \end{array}$$

### 2.6 Preparing chicken burgers

The chicken is purchased at an appropriate amount and about 2 hr after being slaughtered from one of the Faraz Bal Slaughterhouse and transferred in ice‐filled polyester boxes to Orouei meat product laboratory. Chicken meat is scalded and its fats are removed and washed with drinking water. Then, it is grinded with the meat grinder for the tests and kept in the refrigerator until the experiment was carried out. Chicken burger is prepared with the following formulation:

80.23% chicken meat, 11.46% fat, 5.39% water, and 2.92% additive (salt, starch, dextrose, fiber, spices). In order to prepare a chicken burger sample, 800 g of chicken meat was thoroughly cut and grinded by a meat grinder No. 13, then the other ingredients (11 g of oil, 5 g of water, 3 g of additive (salt, starch, dextrose, fiber, spices) were added to the formulation and mixed for 15 min, then the dough was placed in a mold with an arbitrary shape with a length of about 5 cm (Melero, Diez, Rajkovic, Isabel Jaime, & Rovira, 2012). After cutting the chicken burger sample was placed between two films prepared for each treatment and then placed in sterile polyethylene bags. Then, different treatments were kept in a refrigerator at 4°C and microbial tests were performed on days 0, 3, 6, 9, 12. In general, the tests were carried out in six treatments and three replicates.

### 2.7 Chemical analyses

#### 2.7.1 Humidity

To measure moisture, 5 g samples are placed at oven with 105°C for 24 hr (Kalteh, Alizadeh, Dughikla'i, & Yousef Elahi, 2015).

#### 2.7.2 Measuring fat oxidation

Thiobarbituric acid (TBA) was measured by the colorimetric method provided by Egan, Krik, and Sawyer (1997).

### 2.8 Microbigical analyses

#### 2.8.1 Sample preparation for culture

A quantity of 25 g of the sample was weighed in a sterile bag and 225 ml of a 0.14% peptone water diluent solution was added and homogenized with a stomacher for 2 min. From the mixture, a series of dilutions for microbial culture were prepared in the form of 1:10 according to the type of microorganism (Pexara, Metaxopoulos, & Drosinos, 2002).

#### 2.8.2 Counting aerobic mesophilic bacteria

The required dilutions were cultured on Plate Count Agar and incubated for 48 hr at 37°C. Then, the colonies were counted and according to the dilution factor, they were reported as CFU/g (Fernández‐Pan, Carrión‐Granda, & Maté, 2014).

#### 2.8.3 Counting psycrophilic total count

Different dilutions prepared on Plate Count Agar were incubated at 7°C for 7 days and their number was reported as CFU/g according to their dilution factor (Fernández‐Pan et al., 2014).

#### 2.8.4 Counting *P. aeruginosa* bacteria

Different dilutions prepared on Pseudomonas Agar culture medium were incubated at 25°C for 24/84 hr and their number was reported as CFU/g according to the dilution factor (Fernández‐Pan et al., 2014).

#### 2.8.5 Counting *S. aureus* coagulase

Different dilutions prepared on a plate containing Baird Parker Agar Base were incubated at 37°C for 48 hr and then plates containing 20–300 colonies of *S. aureus* were counted. For significant colonies of this microorganism, coagulase test was performed and positive coagulase colonies were reported as *S. aureus*. The coagulase test was performed as follows: the suspicious colonies were transferred to tubes containing 0.3 ml of heart and brain juice and mixed well, and then cultured on a tryptic soy agar diagonal agar using a culture loop. Both the liquid and solid cultures were incubated for 24–18 hr at 37°C. Solid culture was used for storage for confirmatory tests, if necessary. To a culture medium, about 0.5 ml of plasma was added and mixed, the tube containing the culture was re‐incubated at 37°C and were examined for clot formation at intervals of up to 6 hr, and in case of clot formation, 4+ and 3 +  clots were reported as positive coagulase (Downes & Ito, 2000).

#### 2.8.6 Counting *E. coli* O157: H7

Different dilutions prepared on a Palcam Agar medium (containing 5 mg/ml acriflavin and 40 mg/ml nalidixic acid and 40 mg/ml McConkey agar) were incubated at 37°C for 24 hr, and given their dilution factor, they were reported as CFU/g (Shekarforoush, Basiri, Ebrahimnejad, & Hosseinzadeh, 2015).

### 2.9 Sensory evaluation

The sensory quality of the chicken burger samples cooked in the oven at 180°C and 45 min by means of a five‐point hedonic method was investigated by a trained panel of 10 laboratory staff members. Trained panel were asked to judge the odor (5, extremely desirable; 1, extremely unacceptable/off‐odors); and taste (5, extremely desirable; 1, extremely unacceptable) of the samples. The samples were defined as unacceptable when the sensory attributes declined below 4.0 (Ojagh, Rezaei, Razavi, & Hosseini, 2010).

### 2.10 Statistical analysis

The experiments were carried out in three replications in a completely randomized design. Data analysis was done using SPSS statistical software, (release 18.0) for Windows (SPSS Inc. Chicago, IL). One‐way ANOVA was used and mean comparison was performed by Duncans' new multiple range test with a probability level of 5%. Figure was drawn using Microsoft Excel 2013. All data are presented as mean ± SD.

## 3 RESULTS AND DISCUSSION

### 3.1 Investigating the ingredients of AEO

The chemical compounds of the AEO were calculated using the gas chromatography apparatus attached to the mass spectrometer, and the results were presented in Table 1. Among the constituents, 21 combinations containing 99.88% of essential oil compounds were identified. Among the constituents, the most essential compounds were Anethole (74.40%), Thymol (11.44%), Terpinene‐ γ (4.61%), Dl‐Limonene (2.06%), and Estragole (1.87%). The results of this study are consistent with the results of Sharifi, Kiani, Ahmadzadeh, Ahmadzadeh, and Ahmadzadeh (2008) regarding anise essential oil. The most important compounds in their study were E‐anethole (92.9%), p‐allylanisole (2.2%), and Za‐bisabolene (1.8%). In the study of Orav, Raal, and Arak (2008), the major components of the AEO in different regions of Europe were trans‐anethole between 76.9% and 93.7% and γ‐himachalene between 0.4% and 8.2%. In all studies, the most important compounds of AEO were Anethole, and also the studies indicated that the AEO has anti‐bacterial and antioxidant properties and can be used as a natural preservative in the food and pharmaceutical industries. Of course, as you can see, there are differences in the amount and type of essential oil combinations in the various studies, in general, the composition of the herbal essential oils in terms of geographical area, plant variety, age of the plant when preparing the essential oil, environmental and seasonal conditions, type of crop, harvest time, drying and extraction of essential oils, extraction of various organs and eventually genetic variation of the plant can change (Burt, 2004).

**Table 1:** Chemical compounds of anise essential oil (AEO)

Row	Compound name	Duration of inhibition	Percent
1	Anethole	18.363	74.40
2	Thymol	20.113	11.44
3	γ –Terpinene	11.424	4.61
4	Dl‐limonene	10.345	2.06
5	Estragole	16.465	1.87
6	1‐Tetradecene	16.878	1.55
7	Alpha‐thujone	12.460	1.52
8	Dillapiole	30.021	0.74
9	Caryophyllene	23.775	0.72
10	Pulegone	17.865	0.62
11	Beta‐Pinene	8.539	0.18
12	Beta‐Myrcene	9.064	0.17
Total:			99.94

### 3.2 Minimum inhibitory concentration of essential oil

One of the criteria used by most researchers to measure the antibacterial activity of antimicrobial agents is to determine the minimum inhibitory concentration and the minimum concentration of fecundity. Due to the fact that each antimicrobial agent has different effects on microorganisms, this study investigated the AEO on gram‐negative microorganisms such as *S. typhimurium* and *E. coli* and gram‐positive bacteria like *S. aureus* using microdilution method. According to the results (Table 2), the highest MIC values were observed in *Salmonella typhimurium* and the lowest values were found in *S. aureus* (*p* < .05). Several reports have shown that gram‐positive bacteria are more susceptible to antibacterial compounds than gram‐negative bacteria. The resistance of gram‐negative bacteria against antibacterial agents with the hydrophilic surface of the outer membrane of bacteria that is rich in lipopolysaccharide molecules and creates a buffer against the penetration of different antibiotic molecules, as well as with perivascular enzymes that can break the molecules imported from outside are also in contact. Gram‐positive bacteria do not have such an external membrane in the cell wall structure. Some antibiotics can easily destroy the walls of the bacterial cell and the cytoplasmic membrane and lead to the release of its cytoplasm (Shan, Cai, Brooks, & Corke, 2007) Table 2.

**Table 2:** Minimum inhibitory concentration of anise essential oil (AEO)

Treatment	MIC (ppm)
*Escherichia coli* (‐)	4416.66 ± 14.43^b^
*Staphylococcus aureus* (+)	266.33 ± 15.35^c^
*Salmonella* *Typhimurium* (‐)	566.66 ± 14.43^a^

Significant differences in a same column are shown by different letters (*p *<* *.05).

Values are means ± SD (*n* = 3). MIC, minimum growth inhibitor concentration.

### 3.3 Physical characteristics of film

Water sensitivity is one of the major problems of polysaccharide films, which limits their use. The sensitivity of polysaccharide films is measured in various ways such as moisture content, activity and water absorption, solubility and contact angle and water vapor permeability (Bourtoom & Chinnan, 2008). The results for the physical properties of the film are shown in Table 3.

**Table 3:** Evaluation of physical and mechanical characteristics of chitosan (CH) films with anise essential oil (AEO)

Film type	CH	CH + AEO 0.5%	CH + AEO 1%	CH + AEO 1.5%	CH + AEO 2%
Humidity (%)	20.96 ± 0.91^a^	17.99 ± 0.83^b^	17.05 ± 0.15^b^	12.74 ± 0.56^c^	9.91 ± 0.40^d^
Solubility (%)	25.98 ± 0.53^a^	21.66 ± 0.51^b^	16.14 ± 0.25^c^	12.01 ± 0.85^d^	9.49 ± 0.32^e^
Thickness (mm)	0.106 ± 0.004^c^	0.110 ± 0.002^bc^	0.113 ± 0.001^b^	0.122 ± 0.002^a^	0.125 ± 0.003^a^
WVP (×10‐7gs‐1 m‐1 Pa^1^)	3.82 ± 0.07^a^	2.45 ± 0.07^b^	2.27 ± 0.20^b^	1.10 ± 0.02^c^	0.90 ± 0.04^d^
Tensile strength (MPa)	12.66 ± 010^e^	15.85 ± 0.06^d^	16.75 ± 0.56^c^	18.71 ± 0.32^b^	21.38 ± 0.26^a^
Elongation break (%)	5.90 ± 0.03^e^	7.81 ± 0.04^d^	9.24 ± 0.08^c^	10.61 ± 0.35^b^	12.32 ± 0.05^a^

Significant differences in a same row are shown by different letters (*p *<* *.05).

Values are means ± SD (*n* = 3).

The moisture content is a parameter that depends on the total volume occupied by water molecules in the microstructure. The ability to absorb the moisture of a polymer is effective in many features of the film. As by increasing the moisture absorption in the film, the inhibitory properties of it deteriorate (Cuq, Gontard, Aymard, & Guilbert, 1997).

The addition of anise essential oil to chitosan film (20.96%) decreased moisture content, so that the minimum moisture content in chitosan film + AEO was 2% (9.91%) (*p* < .05). This phenomenon is due to the formation of covalent connections between the chitosan chain and the AEO. The formation of these bonding led to the reduction in free hydroxyl and amine groups in the film network and thus reduced the amount of hydrogen bonding between water molecules and the functional groups of polymeric chains, and the reduction in hydrogen bonding resulted in a decrease in the moisture content of films containing anise essential oil (Hosseini et al., 2009).

Water solubility is an important factor in the film that demonstrates the resistance of films against water and is used to package foods that has high water activity, or when films have to be in contact with water and act as food protectors is very important (Bourtoom & Chinnan, 2008). In general, the higher the solubility, the lower the resistance to water (Abdollahi, Rezaei, & Farzi, 2012). The solubility was reduced by adding AEO to chitosan film (25.98%), so that the lowest solubility was observed in chitosan + AEO 2% (9.49%) (*p* < .05). The hydrophobic properties of essential oils, due to their lipid nature, lead to the hydrophobicity of essential oil samples than control film samples and produce a film with lower solubility in water. This type of film can be very useful for covering food that has a wet surface (Bahram et al., 2014).

Thickness is one of the important factors in the film, which directly affects the biological characteristics and durability of the packaged product. According to the results, adding anise essential oil to 1% concentration does not affect the thickness of the film. Increasing the essential oil concentration increased film thickness (*p* < .05). The results of this study correlate with the thickness results of Ojagh et al. (2010) regarding the addition of cinnamon essential oil to chitosan film.

Evaluation of the rate of water vapor permeability of polymer films are absolutely necessary if antimicrobial films are used in foods of medium and high humidity. In order to reduce water vapor permeability, different approaches such as the use of two or more layers of chitosan composites with moisture resistant materials such as hydroxypropylmethyl cellulose (Sebit, Chollet, Degraeve, & Peryorl, 2007) and polymers such as wax starch and another important approach is the use of hydrophobic compounds such as fatty acids, essential oils and extracts. By adding anise essential oil to chitosan film (3.82 × 10^−7^ gs^−1 ^m^−1^ Pa^−1^), the permeability to water vapor decreased, so that the minimum permeability of the film against water vapor was observed in chitosan + anise essential oil 2% (0.90 × 10^−7^ gs^−1 ^m^−1^ Pa^−1^) (*p* < .05). Films with essential oils, due to increased hydrophobicity in the film, decrease the water content of the chitosan film and it acts better in preventing water vapor. These results are consistent with the results of Abdollahi et al. (2012) on the addition of Rosemary essential oil with a concentration of 1.5% to the nanocomposite film and Ojagh et al. (2010) regarding the addition of cinnamon essential oil to chitosan film.

### 3.4 Study of the mechanical properties of films

The mechanical properties of a film such as tensile strength and elongation break determine its ultimate application. Tensile strength is checked through the maximum stress required to tear the film during the tensile test (Noronha, Carvalho, Lino, & Barreto, 2014). The results for the spatial features of the film are shown in Table 3.

The addition of AEO to chitosan film increased the film tensile strength, with increased essential oil concentration, so that the highest increase in film tensile strength was observed in film chitosan + AEO 2% (21.38 MPa). Also, the results of maximum elongation break before the tear point was similar to that of the film tensile strength, so that the highest tensile strengths were observed in the chitosan + AEO 2% (12.32%) (*p* < .05). The reason for this was that a strong interaction between the chitosan polymer and the AEO led to the creation of throughout connections and thus reduced the free volume and molecular mobility of the polymer. This phenomenon leads to the creation of a plate‐like structure and the arrangement of these plates as layers on one another increases the tensile strength (Hosseini et al., 2009). Also, the hydrophilicity and high molecular weight of phenolic compounds of the essential oil, reduce the effects of softening in the film and increase the tensile strength of the essential oil films (Adams, 2007).

### 3.5 Evaluation of moisture content in chicken burger during storage

**Figure 1:**
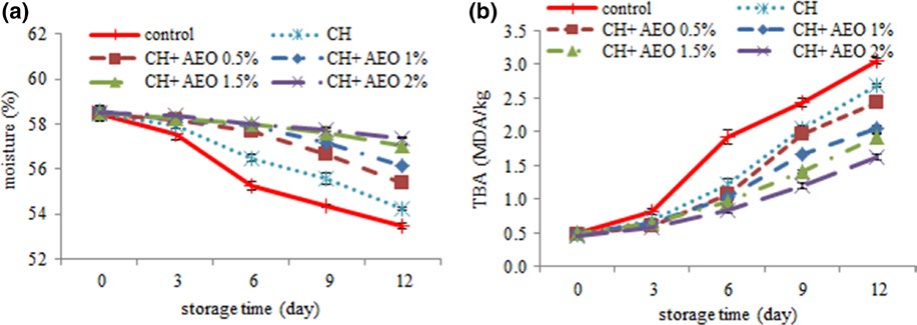
Changes in moisture (a) and thiobarbituric acid (b) of different treatment during storage (C: control, CH: chitosan, CH + 0.5% AEO: chitosan + 0.5% anise essential oil, CH + 1% AEO: chitosan + 1% anise essential oil, CH + 1.5% AEO: chitosan + 1.5% anise essential oil, CH + 2% AEO: chitosan + 2% anise essential oil)

Moisture is one of the important factors in the quality of food. The results of moisture content in this study (Figure 1a) showed that by increasing time, moisture content decreased significantly in all treatments (*p* > .05). The reason for the decrease in moisture content of the samples is the effect of proteolytic enzymes on proteins and their conversion to free amino acids and, consequently, their inability to retain moisture. The reduction in moisture content of the samples, in addition to weight loss, reduces soluble proteins, increases oxidative changes, changes the nature of the protein, changes color and, consequently, reduces product quality (Beklevik, Polat, & Özogul, 2005).

At all times, the lowest moisture content was observed in control treatment, and changes in moisture content were decreased by adding the coating and essential oil. The highest moisture content was observed in chitosan + essential oil (1.5% and 2%) (*p* < .05). In fact, it can be stated that the use of the transfer coating slows down the moisture between the burger and the atmosphere and allows it to control the moisture content of the uncoated treatment. Control of surface moisture content by edible coatings can significantly reduce the growth of microorganisms and the speed of their destructive reactions, thus increasing the storage time of foods (Ozdemir & Floros, 2008).

### 3.6 Investigation of thiobarbituric acid (TBA) changes during storage

In order to evaluate the degree of oxidation of fat in food, the TBA is widely used, indicating the amount of secondary oxidation products, especially aldehydes and ketones (Nishimoto, Suwetja, & Miki, 1985).

With increasing time, the levels of thyobic acid (Figure 1b) increased in all treatments (*p* < .05). The increasing trend of this index is due to the increase in free iron and other peroxides in the muscle, as well as the production of aldehydes from secondary products resulting from the hydroperoxides breakdown (Gomes, Silva, Nascimento, & Fukuma, 2003).

The highest levels of thiobarbituric acid was observed in control treatment. The lowest levels of thyobarbitonic acid were observed in chitosan + essential oil 2%. Generally, biodegradable coatings such as chitosans have a very low permeability to oxygen and carbon dioxide. Thus, the coating formed on the surface of the burgers significantly reduces the contact rate of the product with oxygen, which reduces the rate of initial oxidation of lipids and consequently the formation of hydroperoxides (Mohan, Ravishankar, & Srinivasagopal, 2008). The anise essential oil has the ability to break free radicals by giving a hydrogen atom, and due to its significant amounts of phenolic compounds, the flavonoid has an antioxidant effect that delays oxidative damage in burgers and increased by increasing the percentage of this property in essential oils, and also the combination of essential oil and chitosan caused synergy of antioxidant properties, resulting in better results in chitosan and essential oil 2%. Several studies reported that the antioxidant effect of natural essential oils depends on the amount of their antioxidants (Burt, 2004; Sagdic, 2003). This study also confirms this issue.

The maximum limit for Thiobarbituric of meat fat is 1–2 mg malondialdehyde/kg fat, which is acceptable for human consumption (Lakshmanan, 2000). In this study, chitosan + essential oils 1.5% and 2%, had an acceptable limit until the end of storage period.

### 3.7 Changes in mesophilic bacteria (TVC) in chicken burger during storage

**Figure 2:**
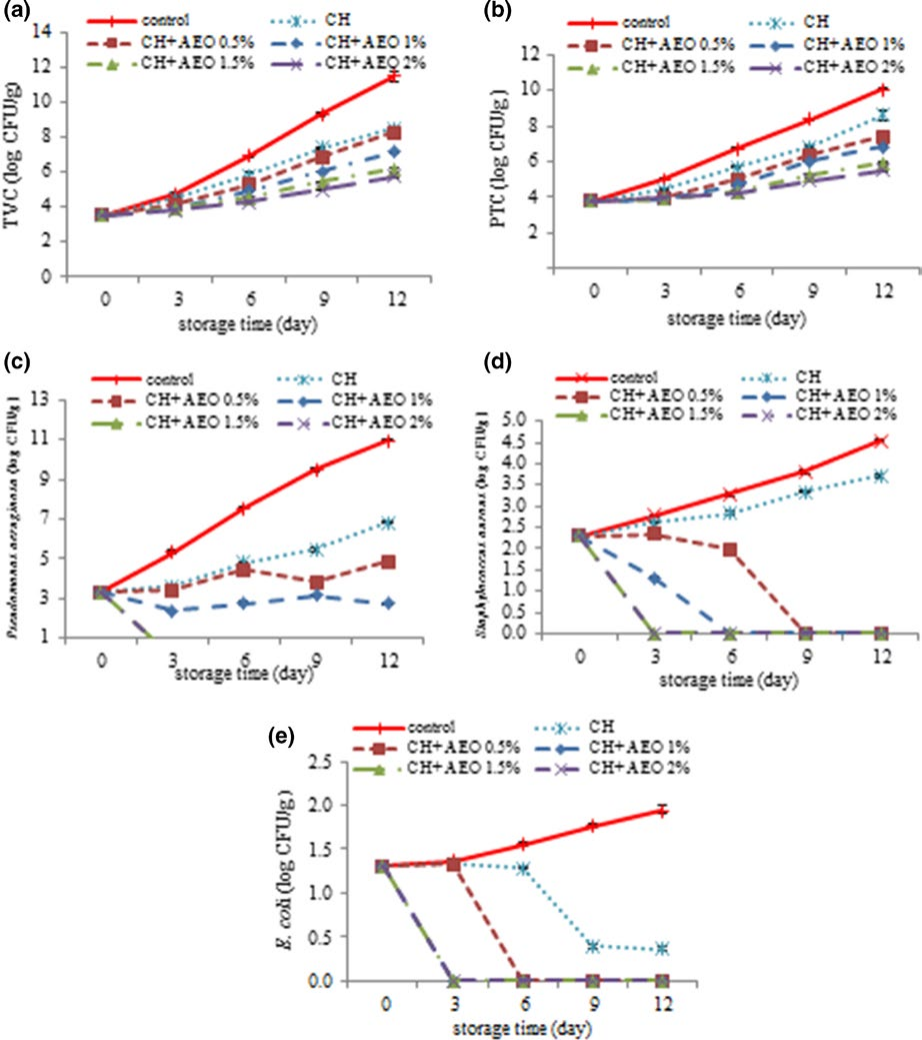
Changes in total viable count (TVC) (a), total psychrotrophic count (TPC) (b), *P. aeruginosa* (c), *S. aureus* (d), *E.coli* (e) of different treatment during storage (C: control, CH: chitosan, CH+ 0.5% AEO: chitosan + 0.5% anise essential oil, CH+ 1% AEO: chitosan+ 1% anise essential oil, CH + 1.5% AEO: chitosan+ 1.5% anise essential oil, CH + 2% AEO: chitosan + 2% anise essential oil)

The total count of microorganisms is a measure to determine the health quality of a product that expresses the nonusability of the product. With increasing time, total viable count (TVC) levels (Figure 2a) increased in all treatments (*p* < .05). The increase in TVC for each treatment during the maintenance period depends on the amount of manipulation, the amount of health in the treatments and the initial rate of bacteria (Chidanandaiah, Keshri, & Sanyal, 2009). According to the results, the addition of chitosan to chicken burger decreased the TVC, which indicates that chitosan coating plays a significant role in reducing the total bacterial load (Chidanandaiah et al., 2009). In all maintenance periods, the lowest amounts of TVC were observed in chitosan + essential oil (2%) (*p* < .05). The low amount of TVC in essential oil treatments can be due to phenolic compounds. The phenolic compounds in plant essential oils of the outer membrane destroy microorganisms and cause the release of liposaccharides and increase the permeability of the cytoplasmic membrane to ATP. The withdrawal of ATP results in the completion of cellular energy storage and cell death (Burt, 2004).

According to the ISRI (2003), the TVC for chicken burger is proposed 6 log CFU/g. In this study, control treatment had a allowed limit up to 4 days and chitosan up to 6 days, chitosan + essential oil 0.5% to 8 days, chitosan + essential oil 1% to 9 days, chitosan + essential oil 1%, chitosan + essential oil, 1.5% to 11 days and chitosan + essential oil 2% up to the end of the maintenance period.

The results of this study in the total bacterium is consistent with results obtained by Ariaii, Tavakolipour, Rezaei, Elhamirad, and Bahram (2015) on the coating of fish fillet with carboxymethylcellulose enriched with *Pimpinella Affinis* essential oil and results of Jalali, Ariiai, and Fattahi (2016), which investigates the effect of combined alginate‐carboxymethyl cellulose coating Clove oil to the amount of TVC filigree fillet.

### 3.8 Investigating the PTC changes in chicken burgers during storage

The main group of microorganisms responsible for the spoilage of freshly stored meat is gram‐negative psycrophilic total count (PTC) (Ojagh et al., 2010). The important characteristics of the PTC is having a strong proteolytic and lipolytic enzyme and their reproductive rate in short time (Sallam, 2007).

In this study, the number of PTC (Figure 2b) increased with increasing storage time. So that the highest amount was observed in control sample and the least in chitosan and essential oil 2% (*p* < .05). The chemical structure of phenolic compounds has an effect on their antimicrobial mechanism. Hydroxyl groups in phenolic compounds have an important role in the antimicrobial activity of essential oils and herbal extracts. The presence of the active hydroxyphenolic group has caused these compounds being able to easily form hydrogen bonds with active sites of enzymes (Burt, 2004). These compounds usually malfunction in the cytoplasmic membrane, break and disrupt the proton movement, electron beam and activated transmission, and cause coagulation of cellular content (Burt, 2004). In fact, the antimicrobial effect of these compounds is due to their reaction with proteins in the cytoplasmic membrane of microorganisms, which changes the permeability of the membrane and the formation of probable pores, and ultimately affects the driving force of the protons (Juven, Kanner, Schved, & Weisslowicz, 1994).

According to ISRI (2003), the authorized PTC for chicken burger as proposed 6 log CFU/g. In this study, control treatment had an allowed limit up to 4 days and chitosan + essential oil 1.5% and 2% until the end of storage period.

### 3.9 Evaluation of changes in *P. aeruginosa* in chicken burger during storage

Pseudomonas is a gram‐negative bacterium, which is one of the major microorganisms responsible for the spoilage of meat. Since the gram‐negative bacteria (and mainly pseudomonas species) are more likely to develop under aerobic and cold conditions, they are the dominant microbial population in meat stored in the refrigerator and exposed to air (Jay, Loessner, & Golden, 2005).

According to the results, in all days, the highest levels of *P. aeruginosa* (Figure 2c) were observed in control treatment (*p* < .05). By adding chitosan, the bacterial levels had a slower rate than the control treatment, which indicates the antimicrobial activity of chitosan. Different mechanisms have been reported for the antimicrobial activity of chitosan: (1) Chitosan inhibits the growth of bacteria as a chelating agent of metallic ions and nutrients, (2) Chitosan is able to bind to ionic groups existing on the surface of bacterial cells and the formation of poly‐electrolyte complexes with compounds present on the surface of the cells of the bacteria, which leads to the lack of transfer of nutrients to the cells and ultimately prevents their growth (Qi, Xu, Jiang, Hu, & Zou, 2004). In treatments containing 0.5 and 1% of AEO, the amount of *P. aeruginosa* was decreasing and increasing. In treatments containing 1.5 and 2% of AEO, from the third day of storage, no values of *P. aeruginosa* were observed. In general, the reduction in bacterial growth in essential oil treatments is due to phenolic compounds in the AEO, which, by increasing the concentration of these compounds, can effectively inhibit bacterial growth and enhance the bactericidal properties. Since the essential oils are mainly lipophilic, they easily pass through the cell wall and the cytoplasmic membrane and disintegrate the structure of different layers of polysaccharides, fatty acids and phospholipids. In bacteria, membrane permeability is associated with loss of ions and membrane potential recovery, proton pump disruption, and reduced ATP reserve. Herbal essential oils can cause the cytoplasm clotting and damaging lipids and proteins. Damage to the wall and membrane of the cell can lead to the evacuation of macromolecules and eventually death of the cell (Bakkali, Averbeck, Averbeck, & Idaomar, 2008).

### 3.10 Evaluation of changes of *S. aureus* in chicken burger during storage

Staphylococcus bacteria are a gram‐positive coccus and β‐hemolytic, which is positive for catalase and coagulase and fermentation of mannitol. This bacterium is commonly the cause of many human infections, and every human being infects the bacterium at least once in his lifetime (Jay et al., 2005). According to the results, over time, the amounts of *S. aureus* (Figure 2d) increased in control and chitosan treatment and the highest levels of bacteria were observed in control treatment (*p* < .05) in treatments containing 0.5% *S. aureus* first, it reduced, and on the ninth day its values reached zero. In the treatment containing 1% of AEO decreased by increasing the time until the third day and on the sixth day the *s. aureus* was kept to zero. In the treatment containing 1.5 and 2% essential oil of the anise from the third day of storage, no values of *S. aureus* was not observed. The antimicrobial activity of anise essential oil against *S. aureus* has also been reported in other researchers (Al‐Bayati, 2008; Gulcin et al., 2003).

According to ISRI (2003), the permitted levels of *Staphylococcus aureus* for chicken burger is 3 log CFU/g. In this study, the control treatment had a permitted limit up to 3 days and the chitosan treatment up to 6 days and the rest of the treatments to the end of the storage period.

### 3.11 Evaluation of changes in *E. coli* bacteria in chicken burger during storage

The *E. coli* bacterium, also called *E. coli*, is a kind of gram‐negative bacilli from the Enterobacteriaceae family. *E. coli* has been identified in many foods worldwide, and the consumption of grinded raw meat is one of the common risks of foodborne pathogenetic infections such as *E. coli* (Solomakos, Govaris, Koidis, & Botsoglou, 2008). Antibiotic resistant *E. coli* are transmitted to humans through the use of meat products and may also cause antibiotic resistance in humans. According to the results, with increasing time, the levels of *E. coli* (Figure 2 e) increased in control treatment. In Chitosan treatments, *E. coli* values were decreasing throughout the maintenance period. In the treatment containing 0.5% AEO, decreased by increasing the time until the third day and on the sixth day, the amount of *E. coli* was reduced to zero. In the treatment containing 1, 1.5, and 2% AEO from the third day of storage, no evidence was found for *E. coli*. ISRI (2003), suggested the permitted levels of *E. coli* for chicken burger log CFU/g negative pathogen. The antimicrobial property of anise essential oil against *E. coli* has been reported by other researchers (Al‐Bayati, 2008; Gulcin et al., 2003).

The results of this study coincided with the results of researchers like Jalali et al. (2016), which indicated that coating treatment containing clove oil 1.5% was able to inhibit the growth of the inoculated Ashirosilicum bacterium in the phytofag fillet and to adhere to the acceptable level.

### 3.12 Sensory evaluation of chicken burger

**Figure 3:**
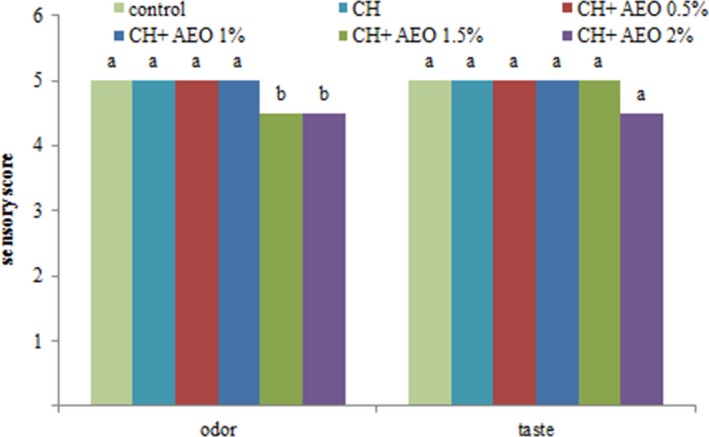
Sensory evaluation of different treatment (C: control, CH: chitosan, CH + 0.5% AEO: chitosan + 0.5% anise essential oil, CH + 1% AEO: chitosan + 1% anise essential oil, CH + 1.5% AEO: chitosan + 1.5% anise essential oil, CH + 2% AEO: chitosan + 2% anise essential oil)

Undoubtedly, sensory characteristics such as odor and taste are the most important factors in accepting a product from a consumer perspective. Therefore, it is important to examine the sensory characteristics by taking into consideration the marketability of the product; sensory analysis is the final guide to the acceptance of the product by the evaluators. According to the results (Figure 3), did not change the chicken odor by adding chitosan and anise essential oil to 1%, but with the increase in AEO concentration, the sensory rating of the chicken burger decreased. Regarding the taste of chicken burger, the addition of chitosan and AEO at 1.5% did not change the taste of chicken burger, but in AEO at 2%, the sensory score decreased. However, all treatments had a sensory score approved by the evaluators. Ojagh et al. (2010) also stated that by adding chitosan and cinnamon essential oil, the sensory analysis of the stored trout fillet decreased as compared to the control treatment. But in sum, all treatments had a sensory rating approved by the evaluators.

## 4 CONCLUSION

In this study, chitosan films containing different concentrations of AEO (0, 0.5, 1, 1.5, and 2%) were used to keep chicken burgers under refrigerated conditions. The results of oxidative spoilage showed that in general chitosan + AEO slowed down the amount of thiobarbituric acid and a decrease in moisture content compared to the control treatment, and a better result was observed with increasing concentrations. The results of microbial studies indicate that in all treatments, the microbial load increased over time, but this increase was observed in chitosan + AEO, and the best results were observed in AEO at 2%. The addition of anise essential oil caused a decrease in *P. aeruginosa*,* S. aureus*,* E. coli*, so that in all treatments containing 1.5 and 2% anise essential oil after 3 days, none of the bacteria was found. Overall, the best results were observed in chitosan + AEO 2%, but this treatment had a lower sensory rating than chitosan + AEO (1.5%). Considering that chitosan + AEO 1.5% and 2% had a proper chemical, microbial and sensory analyzes, and taking into account cost‐effectiveness, the essential oil content 1.5% in film could be an optimal dosage.

## CONFLICT OF INTEREST

None declared.
